# Supplementary material for: Identification of potential anti-tumor targets and mechanisms of HuaChanSu injection using network pharmacology and cytological experiments in Breast cancer
Source: PLoS One. 2024 May 16;19(5):e0303650. doi: 10.1371/journal.pone.0303650 (PMC11098324; doi:10.1371/journal.pone.0303650)
Supplement: Attachment — Submitted filename: Rebuttal letter for P1.docx [file pone.0303650.s001.docx]

**Rebuttal Letter**

**The Affiliated Traditional Chinese Medicine Hospital of Guangzhou Medical University**
No. 16 Zhuji Road, Liwan District
Guangzhou 510130, China
2019620385@gzhmu.edu.cn

October 21, 2023

Dear Editors

We were pleased to have an opportunity to revise our manuscript entitled " *Identification of potential anti-tumor targets and mechanisms of HuaChanSu injection using network pharmacology and cytological experiments in Breast cancer*”. We thank the reviewer for their comments on our manuscript. Below is our response to each point raised by the reviewer. We hope that we satisfyingly addressed them and that the manuscript will be now suited for publication in *PLOS ONE*.

Sincerely,

On behalf of all authors,

Prof. Zhenhuai Yang

**Reviewer 1**

1. **Please check the writing and grammar of the text again.**

We thank the reviewer for this valuable suggestion. English is our second language only, so we have invited native English-speaking editors to revise the manuscript.

2. **Please put the URL address of each database in the Materials and Methods section.**

We apologize for the negligence in our part. We have added the URL address of each database in the Materials and Methods section.

3. **Please edit sentences starting with numbers or abbreviations.**

We thank the reviewer for this valuable suggestion. English is our second language only, so we have invited native English-speaking editors to revise the manuscript.

4. **Why did you not perform cytotoxicity on normal cells? I prefer that this test be done.**

We thank the reviewer for this feedback on our manuscript. We assayed cytotoxic activity of HuaChanSu (HCS) injection to test the antitumor effect of HCS injection, not to observe whether HCS injection is toxic to normal cells. Moreover, normal cells were not used in comparable cytologic studies. So we decided not to perform cytotoxicity on normal cells.

5. **Please specify** **the name and PDB/PubChem ID of the protein targets and ligands in the materials and methods section.**

We thank the reviewer for raising this important issue. As suggested, we have supplemented the name and PDB/PubChem ID of the protein targets and ligands in the Result section (please refer to line 345-358 for the correction).

6. **Please mention how to find the binding site as well as the grid box coordinates in the molecular docking section. In addition, if you intend, you can also calculate the inhibition constant (Ki) based on the binding energy.**

We are grateful for your valuable comments. As suggested, we have supplemented the related information in the materials and methods section (please refer to line 210-215 for the correction).

7. **Please mention on what basis you considered binding energy less than -9 kcal/mol to be stable.**

We sincerely appreciate your valuable comments. According to literature reports, binding energy less than -5.0 kcal/mol is the standard for stable binding of ligands and receptors during molecular docking, and the lower binding energy means the molecular structure is more stable [1]. As you mentioned, we have supplemented the related reference in the materials and methods section (please refer to line 220 for the correction).

8. **Please mention what type of tween** **was used for the western blot.**

We thank the reviewer for pointing this out. As suggested, we have supplemented the related information in the Introductory section (please refer to line 230 for the correction).

9. **Line 207: Please mention what the membrane was washed with.**

We thank the reviewer for constructive comments. As suggested, we have supplemented the related information in the Materials and Methods section (please refer to line 232 for the correction).

10. **Please mention the company, city, and country of the manufacturer of the software.**

We thank the reviewer for careful checks. As the reviewer pointed out, we have supplemented the related information in the Materials and Methods section.

11. **Please increase the resolution of the images. Networks are difficult to evaluate.**

We thank the reviewer for this feedback on our manuscript. We apologize for the inconvenience caused by unclear figures. We have improved the resolution of the figures and re-submitted a new version of the figures.

**Reference**

1.	Gaillard T. Evaluation of AutoDock and AutoDock Vina on the CASF-2013 Benchmark. J. Chem. Inf. Model. 2018;58(8):1697-1706. Epub 2018/07/11. doi: 10.1021/acs.jcim.8b00312. PubMed PMID: 29989806.

**Reviewer 2**

12. **Thank you for stating the following financial disclosure: "The funders had no role in study design, data collection and analysis, decision to publish, or preparation of the manuscript." At this time, please address the following queries:**

**a) Please clarify the sources of funding (financial or material support) for your study. List the grants or organizations that supported your study, including funding received from your institution.**

**b) State what role the funders took in the study. If the funders had no role in your study, please state: “The funders had no role in study design, data collection and analysis, decision to publish, or preparation of the manuscript.”**

**c) If any authors received a salary from any of your funders, please state which authors and which funders.**

a) This work is supported by the Youth Program Research Project of the Affiliated Traditional Chinese Medicine Hospital of Guangzhou Medical University (No. 20210131), Guangzhou Chinese Medicine and Integrative Medicine Science and Technology Project (No. 20232A011005), and Scientific Research Project of Guangdong Provincial Administration of Traditional Chinese Medicine (No. 20221324 and No. 20210078).

b) The funders had no role in study design, data collection and analysis, decision to publish, or preparation of the manuscript.

c) No author receives a salary from any of our funders.

13. **Please note that funding information should not appear in the Acknowledgments section/Funding section or Any other areas of your Manuscript. We will only publish funding information present in the Funding Statement section of the online submission form. Please remove any funding-related text from the manuscript.**

We thank the reviewer for this feedback on our manuscript. We have removed all funding-related text from the manuscript.

14. **PLOS ONE now requires that authors provide the original uncropped and unadjusted images underlying all blot or gel results reported in a submission’s figures or Supporting Information files. This policy and the journal’s other requirements for blot/gel reporting and figure preparation are described in detail at https://journals.plos.org/plosone/s/figures#loc-blot-and-gel-reporting-requirements and https://journals.plos.org/plosone/s/figures#loc-preparing-figures-from-image-files. When you submit your revised manuscript, please ensure that your figures adhere fully to these guidelines and provide the original underlying images for all blot or gel data reported in your submission. See the following link for instructions on providing the original image data: https://journals.plos.org/plosone/s/figures#loc-original-images-for-blots-and-gels. In your cover letter, please note whether your blot/gel image data are in Supporting Information or posted at a public data repository, provide the repository URL if relevant, and provide specific details as to which raw blot/gel images, if any, are not available.**

We thank the reviewer for this valuable suggestion. Our original uncropped and unadjusted blot images were posted at a public data repository (https://www.scidb.cn/s/a6zAzq). We've also revised the cover letter accordingly.

15. **We note your data availability statement as follows: "The datasets used and/or analyzed during the current study are available from the corresponding author on reasonable request." PLOS journals require authors to make all data necessary to replicate their study’s findings publicly available without restriction at the time of publication. PLOS only allows data to be available upon request if there are legal or ethical restrictions on sharing data publicly. (https://journals.plos.org/plosone/s/data-availability) Before we can proceed, please address the following prompts:**

**a.) If there are ethical or legal restrictions on sharing a de-identified data set, please explain them in detail (e.g., data contain potentially identifying or sensitive patient information, data are owned by a third-party organization, etc.) and who has imposed them (e.g., a Research Ethics Committee or Institutional Review Board, etc.). Please also provide non-author contact information* for a data access committee, ethics committee, or other institutional body to which data requests may be sent.**

*** PLOS’ Data Policy states that authors cannot be the sole named individuals responsible for ensuring data access.**

**b) If there are no restrictions, please upload the minimal anonymized or de-identified data set necessary to replicate your study findings to a stable, public repository and provide us with the relevant URLs, DOIs, or accession numbers. For a list of recommended repositories, please see https://journals.plos.org/plosone/s/recommended-repositories. You also have the option of uploading the data as Supporting Information files, but we would recommend depositing data directly to a data repository if possible.**

**We will update your Data Availability statement on your behalf to reflect the information you provide.**

We thank the reviewer for this feedback on our manuscript. Our original uncropped and unadjusted blot images were posted at a public data repository (https://doi.org/10.57760/sciencedb.11831). We've also revised the data availability statement accordingly.
